# Whole-Genome Sequencing for Molecular Characterization of Carbapenem-Resistant Enterobacteriaceae Causing Lower Urinary Tract Infection among Pediatric Patients

**DOI:** 10.3390/antibiotics10080972

**Published:** 2021-08-12

**Authors:** Hassan Al Mana, Sathyavathi Sundararaju, Clement K. M. Tsui, Andres Perez-Lopez, Hadi Yassine, Asmaa Al Thani, Khalid Al-Ansari, Nahla O. Eltai

**Affiliations:** 1 Biomedical Research Center, Qatar University, Doha 2713, Qatar; h.almana@qu.edu.qa (H.A.M.); hyassine@qu.edu.qa (H.Y.); aaja@qu.edu.qa (A.A.T.); 2 Division of Microbiology, Department of Pathology Sidra Medicine, Doha 2713, Qatar; ssundararaju@sidra.org (S.S.); ktsui@sidra.org (C.K.M.T.); aperezlopez@sidra.org (A.P.-L.); 3 Department of Pathology and Laboratory Medicine, Weill Cornell Medical College in Qatar, Doha 2713, Qatar; kalansari@sidra.org; 4 Division of Infectious Diseases, Faculty of Medicine, University of British Columbia, Vancouver, BC V6T 1Z4, Canada; 5 Department of Biomedical Science, College of Health Sciences, QU Health, Qatar University, Doha 2713, Qatar; 6 Department of Emergency, Sidra Medicine, Doha 2713, Qatar

**Keywords:** carbapenem-resistance, *Enterobacteriaceae*, Qatar, CRE, OXA-48

## Abstract

Antibiotic resistance is a growing public health problem globally, incurring health and cost burdens. The occurrence of antibiotic-resistant bacterial infections has increased significantly over the years. Gram-negative bacteria display the broadest resistance range, with bacterial species expressing extended-spectrum β-lactamases (ESBLs), AmpC, and carbapenemases. All carbapenem-resistant *Enterobacteriaceae* (CRE) isolates from pediatric urinary tract infections (UTIs) between October 2015 and November 2019 (*n* = 30). All isolates underwent antimicrobial resistance phenotypic testing using the Phoenix NMIC/ID-5 panel, and carbapenemase production was confirmed using the NG-Test CARBA 5 assay. Whole-genome sequencing was performed on the CREs. The sequence type was identified using the Achtman multi-locus sequence typing scheme, and antimicrobial resistance markers were identified using ResFinder and the CARD database. The most common pathogens causing CRE UTIs were *E. coli* (63.3%) and *K. pneumoniae* (30%). The most common carbapenemases produced were OXA-48-like enzymes (46.6%) and NDM enzymes (40%). Additionally, one *E. coli* harbored IMP-26, and two *K. pneumoniae* possessed mutations in *ompK37* and/or *ompK36*. Lastly, one *E. coli* had a mutation in the *marA* porin and efflux pump regulator. The findings highlight the difference in CRE epidemiology in the pediatric population compared to Qatar’s adult population, where NDM carbapenemases are more common.

## 1. Introduction

Antibiotic resistance is a growing public health problem globally, incurring health and cost burdens. The occurrence of antibiotic-resistant bacterial infections has increased significantly over the years. In 2013, the Center for Disease Control and Prevention (CDC) issued an antibiotic resistance threats report estimating approximately two million infections annually in the United States [1]. By 2017, the number increased to approximately 2.8 million, and deaths increased from 23,000 to 35,900 [2]. Beta-lactams are the most used antibiotics worldwide and include the penicillins, cephalosporins, monobactams, and carbapenems; they all share a typical beta-lactam ring. Gram-negative bacteria display the broadest range of resistance, with bacterial species expressing extended-spectrum β-lactamases (ESBLs), AmpC, and carbapenemases [3]. Of these, carbapenem-resistant *Enterobacteriaceae* (CRE) are of the most concern. Both the CDC and the World Health Organization (WHO) assign CREs the highest urgency levels [2,4].

Carbapenem antibiotics are the choice for treating infections caused by ESBL or AmpC-producing bacteria [5]. Carbapenem resistance is caused mainly by carbapenemase enzymes. Carbapenemase production can be intrinsic, such as Metallo-β-lactamases (MBLs) expressed by *Stenotrophomonas maltophilia*, *Bacillus cereus*, and *Aeromonas* species [6]. In *Enterobacteriaceae*, the most common carbapenemases are categorized into three groups, Ambler classes A, B, and D. The most common group is class A, which includes the *Klebsiella pneumoniae* (*K. pneumoniae)* carbapenemases (KPC) and Imipenem-hydrolyzing β-lactamase (IMI), with KPC being the most prevalent overall [3,7]. Class B contains the Metallo-β-lactamases (MBL), New Delhi Metallo-lactamases (NDM), Imipenem-resistant *Pseudomonas* enzyme (IMP), and the Verona integron-mediated Metallo-lactamase (VIM). Finally, class D contains Oxacillin-hydrolyzing carbapenemases (OXA), of which OXA-48 is the most commonly isolated [3,6]. Carbapenemase genes can be either intrinsic or acquired [8,9]. These carbapenemases are typically plasmid-mediated. In addition to carbapenemases, some members of the *Enterobacteriaceae* family may possess intrinsic carbapenem resistance, arising from mutations in porins or efflux pumps [6].

Several factors increase the risk of CRE infections, including immune suppression, advanced age, intensive care unit (ICU) admission, and previous exposure to antimicrobials [10,11]. *Escherichia coli* (*E. coli*) and *K. pneumoniae* are the most common human pathogens, causing various infections [12,13]. Urinary tract infections (UTIs) are among the most commonly diagnosed infections in children [14]. Empirical treatment for UTIs initially used amoxicillin; however, the increase in resistance of the pathogens, namely *E. coli*, prompted the change to other antibiotics [14]. Recently, with the increased rate of ESBL UTIs, the prescription of carbapenems has increased. However, reports from various regions indicate an increase in the rate of carbapenem resistance [15].

Furthermore, a recently published systematic review, taking into account human, animal, and environmental samples, reported the possibility of the Middle East being an endemic region for CREs [16]. Nevertheless, reports on the prevalence of CREs among the pediatric population, while increasing, are still sporadic [17]. There are limited data describing carbapenem-resistant *Enterobacteriaceae* (CRE) from the Arabian Gulf region among the pediatric population. In Qatar, only one study conducted on different clinical samples from different age groups, presented at the infectious disease forum in 2019, reported that NDM and OXA-48 are the predominant carbapenemases in Qatar and are associated with high overall mortality [18]. The epidemiology of CREs in the adult population is well characterized. As for the pediatric population, while the number of reports is increasing, they remain sporadic, and there is a shortage of data on epidemiology [17]. To that end, we aimed to investigate the genotypic profile of CREs among the pediatric population with UTIs in Qatar.

## 2. Results

### 2.1. Demographics and Etiology

The study of the population’s demographics is summarized in Table 1. The male to female ratio is approximately 1:2.63, with males constituting 27.6% (*n* = 8) and females constituting 72.4% (*n* = 21). CRE infections were more prevalent in non-Qataris (86.2%) compared to Qataris (13.8%). Additionally, 93% of the isolated CREs come from patients aged from 2 months to 13 years. The majority of the CRE UTIs were caused by *E. coli* (*n* = 19, 63.3%), followed by *K. pneumoniae* (*n* = 9, 30%), and the remaining were caused by *Enterobacter hormaechei* (*E. hormaechei*) (*n* = 2, 6.7%). One patient had a mixed infection by *E. coli* and *K. pneumoniae*.

### 2.2. Phenotypic Resistance Profiles of the CRE Isolates

**Figure 1:**
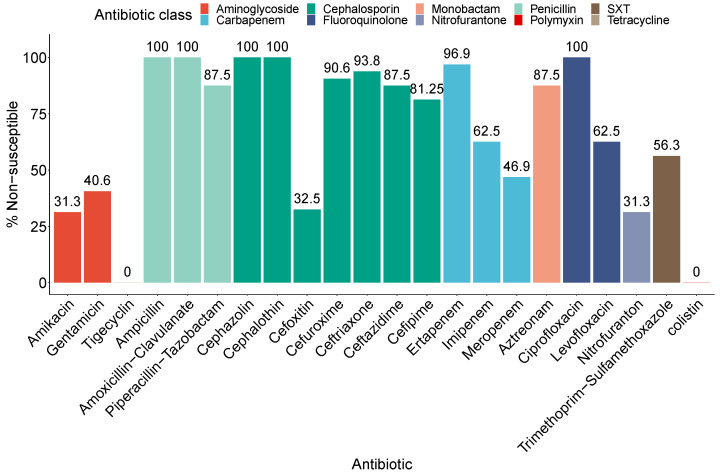
Phenotypic profile of the carbapenem-resistant *Enterobacteriaceae* isolated from children (0–15 years old) with urinary tract infections. The isolates were tested for antibiotic resistance against 22 clinically relevant antibiotics using the Phoenix NMIC/ID-5 panel (BD Biosciences, Heidelberg, Germany). The figure depicts the percentage of isolates that are non-susceptible to each antibiotic. The bars are colored according to the antibiotic class.

The antibiotic resistance profile of all 30 CRE-producing *Enterobacteriaceae* is depicted in Figure 1. All CRE-producing isolates showed 100% resistance to ampicillin, amoxicillin/clavulanate, cefazolin, cephalothin, and ciprofloxacin. High resistance, more than 80%, was detected against cephalosporines, including cefuroxime, ceftriaxone, ceftazidime, and cefepime. High resistance was also noticed against the β-lactam/β-lactamase inhibitor combinations; 87.5% were resistant to piperacillin/tazobactam, whereas 100% were resistant to amoxicillin/clavulanate. All isolates demonstrated resistance to at least one carbapenem, with the highest resistance against ertapenem (96.9%). All isolates were susceptible to colistin and tigecycline. The resistance to other antibiotics, namely, nitrofurantoin, amikacin, cefoxitin, gentamicin, levofloxacin, trimethoprim/sulfamethoxazole, was 31.3%, 31.3%, 32.5%, 40.6%, 62.5%, and 56.3%, respectively.

### 2.3. Molecular Genotyping Profile of CRE Isolates

The genome assemblies included in the study are available at the NCBI website (https://www.ncbi.nlm.nih.gov/bioproject/, accessed on 24 January 2021) under BioProject: PRJNA690895. The accessions for the isolates are detailed in Table S2. The clonal diversity among the isolates was determined; the *E. coli* isolates had 11 different strain types (STs) (ST11021, ST38, ST162, ST448, ST131, ST2083, ST95, ST227, ST410, ST2346, ST10); the *K. pneumoniae* isolates had nine different strain types (ST196, ST45, ST987, ST218, ST101, ST147, ST35, ST870, ST3712); the *E. hormaechei* had the ST 269 and 171 (Table 2).

The antimicrobial markers carried in the isolates are summarized in Table 2 and Table S1. Seven *E. coli* isolates harbored *bla*_OXA-244_, three *E. coli* isolates carried *bla*_NDM-5_, two harbored *bla*_OXA-48_, two fostered *bla*_NDM-4_ while *bla*_NDM-1_, *bla*_IMP-26_, *bla*_OXA-181_, *bla*_OXA-484_, and *marA* mutations were each housed by one *E. coli*. Carbapenem resistance in four *K. pneumoniae* isolates was ascribed to the combination of *bla*_OXA-48/OXA-181/NDM-1_ β-Lactamase production, and porin *ompK36/37* insertional inactivation. Two *K. pneumoniae* harbored *bla*_NDM-1_, one isolate carried *bla*_NDM-5_. Carbapenem resistance in one *K. pneumoniae* was assigned to the *ompK37* mutation and in another isolate is due to the *ompK36* and *ompK37* mutations. *E. hormaechei* harbored *bla*_NDM-1_ and *bla*_NDM-7_ (Table 2).

The locations of the carbapenemases were determined for *E.coli* and *K. pneumonia* using a support vector machine (SVM) algorithm to determine whether the contig of the gene lies on the chromosome or a plasmid. The posterior probabilities of the carbapenemase lying on the plasmid are presented in Table 2. Using a probability cutoff of 0.7 (minimum validated for the algorithm) 21 out of 27 (77%) isolates harbored plasmid type. The algorithm gave no probability for the location of the gene in the two *Enterobacter* isolates as the algorithm was trained on *Enterococcus species*. Only 15.6% (5/32) have other genetic mutations that have been previously associated with carbapenem resistance (Table 2).

## 3. Discussion

CRE UTIs in the pediatric population in Qatar are caused by a diverse set of *E. coli* and *K. pneumoniae* sequence types (STs). The Pediatric Emergency Center sees patients from across the country. The isolates are representative of the general pediatric population. However, no spatial association could be drawn as most patients were not hospitalized, and no information was available on their geographic origin. Moreover, for most isolates, there was no temporal relationship between members of the same species and/or ST except for two isolates (EC-QU-23 and EC-QU-25, Table 2).

CRE infections are associated with mortality rates as high as 65% in the adult population; the rates vary between reports in the pediatric population, with higher rates among neonates [17]. In this study, two patients died within one year of developing CRE UTI (one in 3 months and the other in 11 months). The leading cause of death is attributed to their complicated underlying co-morbidities, namely, congenital heart disease and pulmonary disorders for the first patient and Wiskott–Aldritch for the second.

Carbapenemases are typically carried on plasmids, which enable horizontal gene transfer and their dissemination between bacteria. However, there have been reports of carbapenemases integrated into the chromosome [19]. Multiple isolates showed a probability of the carbapenemase being harbored on the plasmid below the cutoff (0.7). Two isolates, EC-QU-7 and EC-QU-16, had very low probabilities, indicating that the genes are likely on the chromosome. The presence of a carbapenemase on the chromosome can increase dissemination further by allowing vertical transmission while also retaining the ability to transfer horizontally due to the presence of integrons [19].

The most common carbapenemase genes observed in this study are those that encode OXA-48-like carbapenemase lactamases. A previous report on CRE infections in Qatar in the general population (median age 57) and using various sample sources reported NDM-1 and NDM-7 as the most common carbapenemases [18]. However, investigating a sub-population, pediatric UTIs in the study case show a shift in the distribution of carbapenemases. The worldwide dissemination of carbapenemases, particularly in the Middle East, has been suggested due to the importation of CREs through international travel [20]. The contrast in the carbapenemase distribution in this study with the previous report may suggest varying importation sources. The higher prevalence of NDM carbapenemases in that report may be attributed to the higher proportion of migrant workers from the Indian subcontinent, where NDM carbapenemases are endemic [21,22]. However, none of the patients in the study had a recent travel history except for one patient that traveled to India two months before specimen collection (January 2017). The patient had a mixed infection by *E. coli* and *K. pneumoniae* carrying NDM-4 and NDM-1, respectively. Additional information on the carriage rates in the pediatric population and their family members, and their travel history, may provide more concrete data to build a conclusion on.

**Figure 2:**
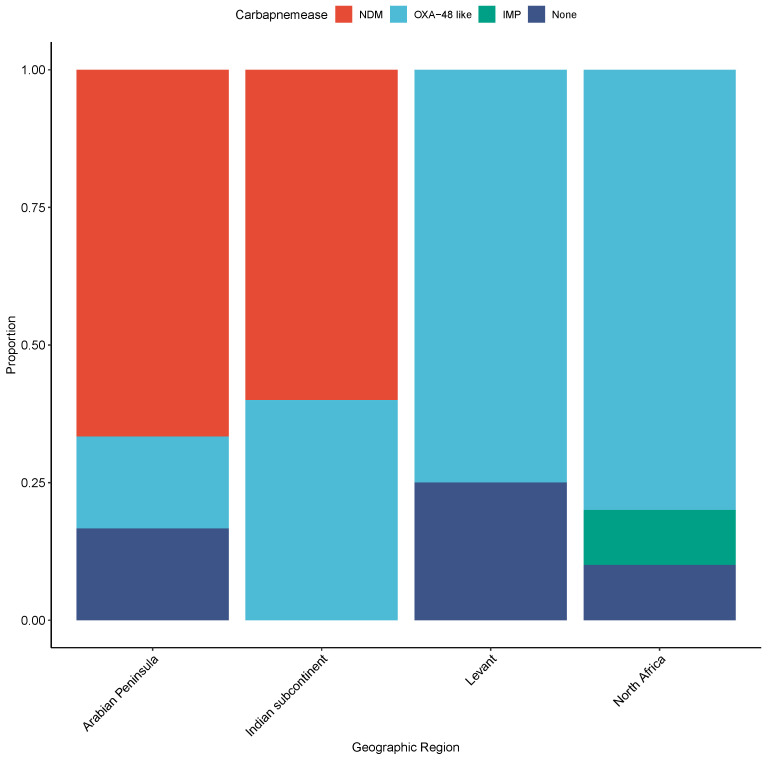
Distribution of carbapenemases by patient ethnicity. Patients from the Arabian Peninsula include the patients from Qatar and the United Arab Emirates. The Levant includes patients from Jordan, Syria, Lebanon, North Africa includes patients from Egypt and Sudan, and the Indian subcontinent includes patients from India and Pakistan.

Nevertheless, there is an apparent relationship between the type of carbapenemase and the nationality of the patient. Of the 12 NDM producers, 66.67% were isolated from patients from the Indian subcontinent (including the patient with travel history to India) compared to 14.3% of OXA-48-like carbapenemase producers (Figure 2). The majority of OXA-48-like enzyme producers were isolated from patients from the Arabian Peninsula, Levant, or North Africa, where OXA-48-like outbreaks or transmission have been reported [23,24].

The most common OXA-48-like carbapenemase observed is OXA-244 (50% of OXA48 like enzymes), followed by OXA-181 and OXA-48. OXA-244 is a single amino acid substitution variant of OXA-48 with a weaker carbapenemase activity that has been frequently reported across Europe [25,26,27]. The lower carbapenemase activity results in lower MICs and more challenging detection, contributing to its silent dissemination [28]. All the isolates carrying OXA-244, all of which were *E. coli*, were non-susceptible to ertapenem and susceptible to imipenem and meropenem, except for one isolate non-susceptible to all three. The *E. coli* isolate that is non-susceptible to all three carbapenems may possess other AMR markers not detected in this study. Notably, all the isolates co-carried a CTX-M type ESBL and/or an AmpC β-lactamase, leading to resistance to third generation cephalosporins. The isolates carrying either carbapenemase were not susceptible to ertapenem or imipenem. Two of the three OXA-181-harboring isolates were from patients whose origin is the Indian subcontinent, which is the main reservoir for this carbapenemase [23].

Three of the isolates did not carry any carbapenemases. One *K. pneumoniae* isolate had mutations in the outer membrane porin OmpK37, and another had mutations in OmpK37 and OmpK36. Modifications of OmpK36/37 contribute to resistance to third generation cephalosporins by reducing antibiotic influx [29,30,31]. However, porin mutations combined with ESBL and/or AmpC β-lactamase have been reported to lead to carbapenem non-susceptibility [32]. The two *K. pneumoniae* isolates carried both an ESBL and AmpC β-lactamase and were non-susceptible to ertapenem and meropenem. The third isolate without a carbapenemase was an *E. coli* with a mutation in the multiple antibiotic resistance A (*marA*) gene. *marA* encodes a transcriptional factor that regulates the expression of multi-drug efflux pumps and porins [33,34,35]. Overexpression of *marA* can result in reduced susceptibility to carbapenems [36]. In addition to *marA*, the isolate also carried several efflux pumps and the DHA-1 AmpC β-lactamase. The combination may potentially explain the observed carbapenem non-susceptibility in a similar mechanism to the porin mutation and ESBL/AmpC combination seen in *K. pneumoniae*.

## 4. Materials and Methods

### 4.1. Clinical Isolates and Control Strains

Institutional Biosafety Committee approval for this study was obtained from the Medical Research Centre (MRC), Hamad Medical Corporation (HMC), Doha, Qatar, protocol no. 16434/16. In total, 30 carbapenem-resistant bacterial isolates were collected between October 2015 and November 2019 from children presented to the Pediatric Emergency Center at Al-Saad, HMC, Qatar, with lower UTIs. All urine analyses were performed on patients who presented with symptoms, mainly fever and dysuria. The urinary catheter was applied for all patients less than or equal to 2 years of age, cerebral palsy (CP) patients, and patients under intermittent catheterization. Otherwise, urine was obtained from the mid-stream catch. Samples that did not yield significant bacterial growth, those with multiple organisms, and samples with suspected contamination as per lab report were excluded from the study, and no duplicate samples were collected. All of the reported cases had UTI as their primary diagnosis. In addition, for each patient, demographic data such as age, nationality, and gender were collected.

*Enterobacteriaceae* species were then isolated using readymade Cystine Lactose Electrolyte-Deficient media (IMES, Doha, Qatar) and identified by MALDI-TOF (Bruker Daltonik GmbH, Leipzig, Germany). Initial antimicrobial susceptibility testing was performed by Phoenix using the NMIC/ID-5 panel (BD Biosciences, Heidelberg, Germany) according to the manufacturer’s recommendations. Briefly, panels were inoculated with 0.5 McFarland pure culture, placed into the instrument, and incubated at 35 °C. The instrument tests the panel every 20 min up to 16 h if necessary. MIC values of each antimicrobial agent are automatically read as susceptible, intermediate, or resistant (SIR). All intermediates were considered susceptible, and susceptibility testing was performed for twenty-two clinically relevant antibiotics. Both automated tests were performed at Hamad General Hospital Microbiology laboratory. Thirty-two out of 36 detected carbapenem-resistant isolates were subjected to further genotypic analysis (strain characteristics of these isolates are described in Table 1), and non-*Enterobacteriaceae* were excluded.

Standard strains, *E. coli* ATCC^®^ 25922™ and *E. coli* ATCC^®^ 35218™ were used as controls for antimicrobial drug susceptibility testing. *Enterobacter cloacae* complex.

NCTC^®^ 13925^TM^, *K. pneumoniae* NCTC^®^ 13440^TM^, *K. pneumoniae* NCTC^®^ 13443^TM^, *E. coli* NCTC^®^ 13476 ^TM^ IMP, and *K. pneumoniae* NCTC^®^ 13442^TM^ were used as positive controls for class A carbapenemases (IMI), class B Carbapenemases (Metallo-β-lactamases: VIM-1, NDM-1, IMP) and class D Carbapenemases (OXA-48), respectively. All intermediate-resistant isolates were considered susceptible. These clinical isolates were preserved at −80 °C for further analysis.

### 4.2. Carbapenemase Phenotypic Confirmation

The isolates resistant to meropenem, ertapenem, or imipenem were further tested with the NG-Test CARBA 5 assay (NG Biotech, Guipry, France), following the manufacture’s protocol. The assay detects the presence of NDM, VIM, IMP, KPC, and OXA-48-like carbapenemases. In the event that an isolate was negative for the five carbapenemases, it was tested with both ertapenem and meropenem E-tests (Liofilchem, Roseto degli Abruzzi TE, Italy) and interpreted following the Clinical Laboratory Standards Institute’s (CLSI) guidelines.

### 4.3. Molecular Characterization

DNA extraction, whole-genome sequencing, and bioinformatic analysis.

Genomic DNA was extracted from all *E. coli*, *K. pneumoniae*, and *Enterobacter cloacae* (*E. cloacae*) isolates that were positive for one of the five carbapenemase genes. Extraction was performed using the QIAamp^®^ UCP Pathogen mini kit (Qiagen, Düsseldorf, Germany) following the manufacturer’s protocol. Briefly, genomic DNA was purified from each isolate and later quantified using a Qubit dsDNA high sensitivity assay (Thermo Fisher, Waltham, MA, USA). Whole-genome sequencing was performed on the Illumina MiSeq platform, using Nextera XT (Illumina, San Diego, CA, USA), for paired-end (PE) library construction; the DNA was tagmented, amplified with index primers, and purified with AMPure XP beads (Beckman Coulter, Brea, CA, USA). Finally, the DNA library was normalized, pooled, and sequenced using the MiSeq platform with 300bp PE reads (MiSeq Reagent Kit V3). The raw sequences were subjected to a quality check and analyzed using CLC genomics workbench v20.0.4 (https://digitalinsights.qiagen.com; accessed on 20 August 2020). Briefly, the reads were quality assessed, trimmed, and followed by de novo assembly. Resistance genes were identified using ResFinder v 4.1 [37] and CARD’s comprehensive antibiotic resistance database [38]. Only resistance genes that showed a perfect match, with 100% identity and coverage for a given gene in the database, were reported in this study. Sequence type was identified by an exact match against the chosen locus scheme Achtman via pubMLST database for molecular typing (www.pubMLST.org accessed on 20 August 2020). Identification of mobile genetic elements, and their relation to antibiotic resistance were analyzed through Mobile Element Finder [39], and the location of the gene was determined using mlplasmids v1.0.0 [40]. In brief, mlplasmid uses SVM algorithm pentamer frequencies to classify the contigs harboring the genes of interest as plasmid- or chromosome-derived using maximum likelihood.

## 5. Conclusions

The most common carbapenemases in pediatric UTIs in Qatar are OXA-48-like carbapenemases. CREs expressing these carbapenemases may pose a threat of silent transmission as they typically confer low-level resistance to carbapenems, particularly in the case of OXA-224. Additionally, the presence of CREs that gain resistance through mechanisms other than carbapenemases highlights the limitation of the common screening methods that rely only on the presence of carbapenemases. While the sample is comprehensive, including all CRE UTIs over the period between October 2015 and November 2019, the small sample size and lack of all-inclusive clinical information preclude in-depth analysis of the clinical manifestations and implications on the treatment of such infections or measuring meaningful associations. Nevertheless, the results warrant further investigation on the epidemiology of CRE infections in the pediatric population, particularly in terms of carriage, to elucidate the distribution and transmission dynamics of CREs.

## Figures and Tables

**Table 1:** Demographic profile of the study population (*n = 29*).

Gender	Total Number (%)	Nationality	
		Qatari	Non-Qatari
Male	8 (27.6%)	0 (0%)	8 (27.6%)
Female	21 (72.4%)	4 (13.8%)	17 (58.6%)
Total	29 (100%)	4 (13.8%)	25 (86.2%)
Age			
<2	5 (17.2%)	0 (0%)	5 (17.2%)
2–5	13 (44.8%)	1 (3.4%)	12 (41.4%)
6–15	11 (37.9%)	3 (10.3%)	8 (27.6%)
Total	29 (100%)	4 (13.8%)	25 (86.2%)

**Table 2:** Antibiotic resistance genes present in the isolates.

Isolate ID	Collection Date (Month-Year)	Sequence Type (ST)	Carbapenem Resistance Genes (Mlplasmid Posterior Probability for Carbapenemases)
*Escherechia coli*			
EC-QU-7	June-2015	162	*bla*_NDM-1_ *(0.126)*
EC-QU-5	November-2015	38	*bla_OXA-48_ (0.971)*
EC-QU-14	November-2015	448	*bla_NDM-5_ (0.63)*
EC-QU-1	April-2016	11,021	*bla_NDM-4_ (0.937)*
EC-QU-10	December-2016	11,021	*marA* mutation
EC-QU-16	January-2017	131	*bla_IMP-26_ (0.126)*
EC-QU-6	March-2017	11,021	*bla_NDM-4_ (0.764)*
EC-QU-19	September-2018	2083	*bla_NDM-5_ (0.65)*
EC-QU-21	October-2018	162	*bla_NDM-5_ (0.79)*
EC-QU-23	January-2019	38	*bla_OXA-244_ (0.852)*
EC-QU-25	January-2019	38	*bla_OXA-244_ (0.972)*
EC-QU-26	May-2019	38	*bla_OXA-244_ (0.80)*
EC-QU-27	July-2019	95	*bla_OXA-181_ (0.84)*
EC-QU-31	August-2019	410	*bla_OXA-484_ (0.78)*
EC-QU-30	September-2019	227	*bla_OXA-48_ (0.94)*
EC-QU-29	September-2019	38	*bla_OXA-244_ (0.991)*
EC-QU-28	September-2018	131	*bla_OXA-244_ (0.94)*
EC-QU-33	September-2019	2346	*bla_OXA-244_ (0.983)*
EC-QU-35	October-2019	10	*bla_OXA-244_ (0.731)*
*Klebsiella pneumonaie*			
KPN-QU-9	October-2015	45	*ompK37* mutation
KPN-QU-15	January-2017	218	*ompK37 and ompK36* mutations
KPN-QU-17	March-2017	101	*bla_NDM-1_ (0.884)*
KPN-QU-3	March-2017	196	*bla_NDM-1_ (0.992)*
KPN-QU-11	April-2017	987	*ompK37* mutation *bla_OXA-48_ (0.947)*
KPN-QU-20	April-2018	147	*bla_NDM-5_ (0.942)*
KPN-QU-22	January-2019	35	*ompK37, ompK36* mutations *bla_OXA-181_ (0.913)*
KPN-QU-37	August-2019	3712	*ompK37* mutation *bla _OXA-181_ (0.97)*
KPN-QU-36	October-2019	870	*ompK37 and ompK36* mutations *bla_NDM-1_ (0.982)*
*Enterobacter hormaechi*			
EBH-QU-2	October-2016	269	*bla_NDM-7_* ^a^
*Enterobacter cloacae*			
EBH-QU-4	December-2016	171	*bla_NDM-1_* ^a^

^a^ Posterior probability was not determined for these isolates.

## Data Availability

The genome assemblies included in the study are available at the NCBI website (https://www.ncbi.nlm.nih.gov/bioproject/, accessed on 24 January 2021) under BioProject: PRJNA690895. The accessions for the isolates are detailed in Table S2.

## Supplementary Materials

The following are available online at https://www.mdpi.com/article/10.3390/antibiotics10080972/s1, Table S1: Non-β-lactamase antimicrobial resistance genes present in the isolates, Table S2: Sample assembly accessions.
