# Supplementary material for: Whole-Genome Sequencing for Molecular Characterization of Carbapenem-Resistant Enterobacteriaceae Causing Lower Urinary Tract Infection among Pediatric Patients
Source: Antibiotics (Basel). 2021 Aug 12;10(8):972. doi: 10.3390/antibiotics10080972 (PMC8388976; doi:10.3390/antibiotics10080972)
Supplement: Supplementary file 1 [file antibiotics-10-00972-s001.zip › antibiotics-1287019-supplementary.pdf]

Table S1. Non-β-lactamase antimicrobial resistance genes present in the isolates

Isolate ID	β-lactamase genes	Fluoroquinolone resistance markers	Aminoglycoside resistance markers	Co-trimethoxazole resistance markers	Tetracycline resistance markers	Other resistance markers
EC-QU-1	bla_{TEM-1B}	*gyrA*	*rmtB*	*sul1, dfrA17, dfrA12*	*tetA*	*mph(A)*
EBH-QU-2	$bla_{CTX-M-15}$, bla_{CMY-42}	*qnrS, aac(6′)-Ib-cr*	*aac(6′)-Ib-cr*	*sul1, dfrA14*		
KPN-QU-3	$bla_{CTX-M-15}$, bla_{TEM-1A}, bla_{OXA-9}, bla_{CMY-42}, bla_{ampC}	*oqxB, qnrS1, aac(6′)-Ib-cr*	*aac(6′)-Ib-cr*	*sul1, dfrA14*		
EBH-QU-4	bla_{OXA-1}	*qnrB1, aac(6′)Ib-cr*	*rmtC, aac(3)-Iia, aac(6′)-Ib-cr*	*dfrA14*		
EC-QU-5	$bla_{CTX-M-99}$, $bla_{CTX-M-122}$, $bla_{CTX-M-, 196}$, $bla_{CTX-M-125}$, $bla_{CTX-M-14}$, $bla_{CTX-M-65}$, bla_{TEM-1B}	*gyrA*	*aac(3)-Iid*	*sul1*		*mph(A)*
EC-QU-6	bla_{TEM-1B}, bla_{CMY-42}	*gyrA, parC*	*rmtB, aadA5, aadA2*	*sul1, dfrA17, dfrA12*	*tetA*	*mph(A)*
EC-QU-7	$bla_{CTX-M-15}$, bla_{TEM-1A}, bla_{OXA-9}, bla_{CMY-42}, bla_{ampC}	*gyrA, aac(6′)-Ib-cr, qnrS1*	*aac(6)-Ib, aph(3)-VI, aac(6′)-Ib-cr*			
KPN-QU-9	bla_{SHV-98}, bla_{SHV-26}, bla_{SHV78}, bla_{ACT-7}	*acrR*		*sul1, dfrA25*	*tetA*	*fosA*
EC-QU-10	bla_{DHA-1}	*gyrA, parC, mdf(A)*				
KPN-QU-11	bla_{SHV-27}	*acrR*				
EC-QU-14	bla_{TEM-1B}, bla_{OXA-1}, bla_{ACT-16}	*aac(6′)-Ib-cr*	*rmtB, aac(3)-Iid, aac(6′)-Ib-cr*	*sul1, dfrA17, dfrA12*		*mph(A)*
KPN-QU-15	$bla_{CTX-M-15}$, bla_{TEM-1B}, bla_{SHV-75}, bla_{SHV-33}, bla_{SHV-51}, bla_{ampC}	*acrR, oqxA, oqxB*	*aph(3)-Ib, aph(6)-id*	*sul2, dfrA14*		*fosA*
EC-QU-16	bla_{OXA-1}	*gyrA, aac(6′)-Ib-cr*	*aac(6′)-Ib3. aac(6′)-Ib-cr*	*sul1, dfrA17*		*mph(A)*

KPN-QU-17	*bla*$_{CTX-M-15}$, *bla*$_{SHV-106}$, *bla*$_{TEM-1B}$, *bla*$_{ampC}$	*aac(6′)-Ib-cr, oqxA, oqxB*	*aac(6′)-Ib-cr, aac(6′)-Ib-Hangzhou, rmtF*	*sul2*		
EC-QU-19	*bla*$_{TEM-1B}$	*gyrA, aac(6′)-Ib-cr*	*aac(6′)-Ib-cr, rmtB*	*sul1, dfrA12*	*tetB*	
KPN-QU-20	*bla*$_{CTX-M-15}$, *bla*$_{TEM-1B}$, *bla*$_{SHV-11}$, *bla*$_{SHV-67}$, *bla*$_{OXA-1}$, *bla*$_{OXA-9}$, *bla*$_{DHA-1}$	*gyrA, aac(6′)-Ib-cr, oqxA, oqxB, qnrS1, acrR*	*aac(6′)-Ib-cr, rmtB, aadA1, aadA2*	*sul1, dfrA12*	*tetB*	*fosA*
EC-QU-21	*bla*$_{CTX-M-15}$, *bla*$_{TEM-207}$, *bla*$_{TEM-1B}$, *bla*$_{TEM-230}$, *bla*$_{ampC}$	*qnrS1*	*rmtB*	*sul1*		
KPN-QU-22	*bla*$_{SHV-98}$, *bla*$_{SHV-31}$, *bla*$_{SHV-86}$, bla$_{SHV-13}$, *bla*$_{SHV-129}$, *bla*$_{SHV-12}$, *bla*$_{SHV-61}$	*oqxA, oqxB, qnrS1*	*aadA1*	*sul1, dfrA1*	*tetA*	
EC-QU-23	*bla*$_{CTX-M-27}$, *bla*$_{ampC}$	*mdf(A)*	*aph(6)-Id, aph(3)-Ib, aadA5*		*tetA*	*mph(A)*
EC-QU-25	*bla*$_{CTX-M-14b}$, *bla*$_{ampC}$	*emrR, emrB, mdtH, mdtF*				
EC-QU-26	*bla*$_{CTX-M-15}$, *bla*$_{TEM-1B}$, *bla*$_{CMY-2}$, *bla*$_{ampC}$	*qnrS1*		*sul1, sul2, dfrA17*		
EC-QU-27	*bla*$_{TEM-1B}$, *bla*$_{ampC}$	*acrB, mdtH, evgA, emrR*				
EC-QU-28	*bla*$_{ampC}$	*gyrA*		*sul1*		*mph(A)*
EC-QU-29	*bla*$_{CTX-M-27}$, *bla*$_{CMY-42}$	*mdtF, mdtH, marA, emrA, emrB, evgA*	*aph(3′′)-Ib, aph(6)-Id*	*sul1, sul2. dfrA17*	*tetA*	
EC-QU-30	*bla*$_{CTX-M-15}$, *bla*$_{ampC}$	*qnrS*		*sul1, dfrA17*	*tetA*	*mph(A)*
EC-QU-31	*bla*$_{TEM-1B}$, *bla*$_{ampC}$	*qnrS1*	*aph(3′′)-Ib, aadA5*	*sul1, sul2*		*mph(A)*
EC-QU-33	*bla*$_{CTX-M-15}$, *bla*$_{TEM-1}$, *bla*$_{ampC}$	*qnrS1*	*aadA1*	*sul2, dfrA14*		
EC-QU-35	*bla*$_{TEM-1B}$, bla$_{CMY-42}$, *bla*$_{ampC}$		*aph(3′′)-Ib, aph(6)-Id*	*sul2, dfrA14*	*tetA*	

KPN-QU-36	*bla*$_{CTX-M-15}$, *bla*$_{SHV-106}$, *bla*$_{TEM-1B}$, *bla*$_{OXA-1}$, *bla*$_{CMY-6}$	*oqxA, oqxB, aac(6′)-Ib-cr, acrR*	*rmtC, aph(3′)-Ia, aac(6′)-Ib-cr, aph(6)-Id*	*sul1*	
KPN-QU-37	*bla*$_{SHV-33}$, *bla*$_{ampC}$	*acrR, soqxA, oqxB, qnrS1, qnrB4*	*aph(3′′)-Ib, aph(6)-Id*	*sul1*	*fosA*

Table S2. Sample assembly accessions.

Sample ID	NCBI Strain	BioProject	BioSample	Accession	Assembly accession
EC-1	QU-1	PRJNA690895	SAMN17315638	JAFBLD000000000	GCA_016809875.1
ENH-2	QU-2	PRJNA690895	SAMN17320769	JAFBKQ000000000	GCA_016810215.1
KPN-3	QU-3	PRJNA690895	SAMN17320971	JAFBKT000000000	GCA_016810295.1
ENC-4	4	PRJNA690895	SAMN17267145	JAFBKT000000000	GCA_016745405.1
EC-5	QU-5	PRJNA690895	SAMN17315802	JAFBKB000000000	GCA_016809855.1
EC-6	QU-6	PRJNA690895	SAMN17320896	JAFBKR000000000	GCA_016810195.1
EC-7	QU-7	PRJNA690895	SAMN17315989	JAFBKC000000000	GCA_016809895.1
KPN-9	QU-9	PRJNA690895	SAMN17320972	JAFBKU000000000	GCA_016810255.1
EC-10	QU-10	PRJNA690895	SAMN17320938	JAFBKS000000000	GCA_016810235.1
KPN-11	QU-11	PRJNA690895	SAMN17348687	JAFBKV000000000	GCA_016810895.1
EC-14	QU-14	PRJNA690895	SAMN17316100	JAFBKD000000000	GCA_016809905.1
KPN-15	QU-15	PRJNA690895	SAMN17348775	JAFBKW000000000	GCA_016810915.1
EC-16	QU-16	PRJNA690895	SAMN17316153	JAFBKE000000000	GCA_016809935.1
KPN-17	QU-17	PRJNA690895	SAMN17348776	JAFBKX000000000	GCA_016810935.1
EC-19	QU-19	PRJNA690895	SAMN17319936	JAFBKF000000000	GCA_016809955.1
KPN-20	QU-20	PRJNA690895	SAMN17348778	JAFBKY000000000	GCA_016810965.1
EC-21	QU-21	PRJNA690895	SAMN17319937	JAFBKG000000000	GCA_016809975.1
KPN-22	QU-22	PRJNA690895	SAMN17348779	JAFBKZ000000000	GCA_016810995.1
EC-23	QU-23	PRJNA690895	SAMN17320764	JAFBKP000000000	GCA_016810175.1
EC-25	QU-25	PRJNA690895	SAMN17319964	JAFBKH000000000	GCA_016809995.1
EC-26	QU-26	PRJNA690895	SAMN17320328	JAFBKI000000000	GCA_016810035.1

EC-27	QU-27	PRJNA690895	SAMN17320627	JAFBKJ000000000	GCA_016810015.1
EC-28	QU-28	PRJNA690895	SAMN17320630	JAFBLC000000000	GCA_016810055.1
EC-29	QU-29	PRJNA690895	SAMN17320631	JAFBKK000000000	GCA_016810075.1
EC-30	QU-30	PRJNA690895	SAMN17320633	JAFBKL000000000	GCA_016810095.1
EC-31	QU-31	PRJNA690895	SAMN17320761	JAFBKM000000000	GCA_016810115.1
EC-33	QU-33	PRJNA690895	SAMN17320762	JAFBKN000000000	GCA_016810135.1
EC-35	QU-35	PRJNA690895	SAMN17320763	JAFBKO000000000	GCA_016810155.1
KPN-36	QU-36	PRJNA690895	SAMN17348780	JAFBLA000000000	GCA_016810955.1
KPN-37	QU-37	PRJNA690895	SAMN17348781	JAFBLB000000000	GCA_016811015.1
